# Localized electron transfer rates and microelectrode-based enrichment of microbial communities within a phototrophic microbial mat

**DOI:** 10.3389/fmicb.2014.00011

**Published:** 2014-01-27

**Authors:** Jerome T. Babauta, Erhan Atci, Phuc T. Ha, Stephen R. Lindemann, Timothy Ewing, Douglas R. Call, James K. Fredrickson, Haluk Beyenal

**Affiliations:** ^1^The Gene and Linda Voiland School of Chemical Engineering and Bioengineering, Washington State UniversityPullman, WA, USA; ^2^Biological Sciences Division, Pacific Northwest National LaboratoryRichland, WA, USA; ^3^Paul G. Allen School for Global Animal Health, Washington State University College of Veterinary MedicinePullman, WA, USA

**Keywords:** electron transfer, hot lake, microbial mats, microelectrodes, sulfur cycle, sequence analysis

## Abstract

Phototrophic microbial mats frequently exhibit sharp, light-dependent redox gradients that regulate microbial respiration on specific electron acceptors as a function of depth. In this work, a benthic phototrophic microbial mat from Hot Lake, a hypersaline, epsomitic lake located near Oroville in north-central Washington, was used to develop a microscale electrochemical method to study local electron transfer processes within the mat. To characterize the physicochemical variables influencing electron transfer, we initially quantified redox potential, pH, and dissolved oxygen gradients by depth in the mat under photic and aphotic conditions. We further demonstrated that power output of a mat fuel cell was light-dependent. To study local electron transfer processes, we deployed a microscale electrode (microelectrode) with tip size ~20 μm. To enrich a subset of microorganisms capable of interacting with the microelectrode, we anodically polarized the microelectrode at depth in the mat. Subsequently, to characterize the microelectrode-associated community and compare it to the neighboring mat community, we performed amplicon sequencing of the V1–V3 region of the 16S gene. Differences in Bray-Curtis beta diversity, illustrated by large changes in relative abundance at the phylum level, suggested successful enrichment of specific mat community members on the microelectrode surface. The microelectrode-associated community exhibited substantially reduced alpha diversity and elevated relative abundances of *Prosthecochloris*, *Loktanella, Catellibacterium*, other unclassified members of *Rhodobacteraceae*, *Thiomicrospira*, and *Limnobacter*, compared with the community at an equivalent depth in the mat. Our results suggest that local electron transfer to an anodically polarized microelectrode selected for a specific microbial population, with substantially more abundance and diversity of sulfur-oxidizing phylotypes compared with the neighboring mat community.

## INTRODUCTION

Microbial mats are highly stratified microbial communities where metabolically interacting species inhabiting strata generated by sharp physicochemical gradients transfer energy via metabolic byproducts (Franks and Stolz, 2009). The proximity and density of organisms inside microbial mats makes energy transfer highly efficient, where energy transfer refers to oxidation–reduction reactions that predominantly cycle carbon and sulfur throughout the mat. These properties make microbial mats exceptional candidates for specific applications, including carbon sequestration and wastewater treatment (Roeselers et al., 2008). Using microbial mats in this manner requires precise control of the physicochemical gradients that drive energy transfer where one possibility to control such gradients is to electrochemically change the mat environment by providing (reduction) or removing (oxidation) electrons. Theoretically, this could be accomplished inside the microbial mat by using electrodes. Because organisms in the mat proliferate within spatially defined niches along physicochemical gradients in the mat, drawing a current is likely to cause changes in niche localization and hence alter the composition of the local microbial community. Therefore, quantifying the effect of electron transfer within microbial mats could also be used to understand the energy balance that leads to stratification of the species and associated functions within the mat.

Microbial mats are similar to microbial biofilms, except that microbial mats exhibit lamination (Guerrero et al., 2002). Biofilms tend to be composed of a single species and form readily on electrode surfaces, making them popular model systems in which to study electron transfer mechanisms. In contrast, a high diversity of species and, therefore, microbial metabolisms (e.g., photosynthesis, sulfate reduction) simultaneously operate at various locations within a mat. Consequently, efforts to detect electron transfer in the mat need to be spatially precise and take into account dynamic physicochemical gradients. Understanding local electron transfer within a mat is made increasingly difficult because the diversity and dynamicity of metabolism generates a complex web of metabolites that shifts over time. Therefore, the common method of employing macro-scale, passive microbial fuel cells to detect power generation by either anodic or cathodic microorganisms that respond to the microbial fuel cell is not capable of directly measuring local electron transfer rates *inside* a mat. However, several studies have demonstrated microbial fuel cells that use a photosynthetic community colonized on the electrodes to produce power (He et al., 2009; Nishio et al., 2010; Bradley et al., 2012; Chandra et al., 2012; Badalamenti et al., 2013; Lan et al., 2013; Lin et al., 2013; Strycharz-Glaven et al., 2013). However, power generation is not our focus here. These techniques are limited because all use large electrodes and therefore do not possess the resolution to measure local electron transfer processes in stratified systems at the microscale. Our goal was to determine if local electron transfer inside a mat could be measured and, if so, manipulated to induce changes in the local community.

Local electron transfer can be resolved by scaling down to needle-type microscale electrodes, or simply microelectrodes (Lewandowski and Beyenal, 2014). Initially, when a microelectrode with a carbon tip is inserted into a desired location in a microbial mat, the microelectrode tip responds to redox couples and reaches an open circuit potential. Once the microelectrode tip is polarized against a reference electrode, an electrochemical gradient is generated where electroactive compounds can be oxidized or reduced. Subsequently, some bacteria will respond to an increase/decrease in electroactive compounds while other species should be able to take advantage of the electrochemical gradient directly by transferring electrons to the electrode, as happens with known dissimilatory metal-reducing bacteria (Shi et al., 2009; Borole et al., 2011; Babauta et al., 2012a). However, we note that known dissimilatory metal-reducing phylotypes (e.g., *Geobacter*, *Shewanella*) were not detected within the Hot Lake mat community over one annual cycle (Lindemann et al., 2013). Regardless of the specific mechanism, the electrochemical gradient should only cause a change if current is generated; making local current generation a useful indicator to determine if a community change has occurred. In the absence of current generation, polarization of the microelectrode tip could also cause physical association of microorganisms considering van der Waals and electrostatic interactions (Hori and Matsumoto, 2010). However, the changes in electrostatic interactions caused by charging/discharging of the electric double layer at the electrode surface is generally neutralized by ions in solution at the overpotentials used here and given the high ionic strength of Hot Lake water.

Although there is a long history of microscale measurements made in biofilms and microbial mats, these studies have not been performed with polarized microelectrodes (Jørgensen, 1983; Revsbech and Ward, 1984; de Beer et al., 1994; Kühl et al., 1994; Beyenal et al., 1998; Bishop and Yu, 1999; Nguyen et al., 2012). Earlier studies focused on the penetration of oxygen in biofilms and microbial mats, which allowed microaerophilic and anaerobic modes of life to exist even in oxic waters. In addition to biological consumption of oxygen, we have characterized the electrochemical consumption of oxygen and other relevant species within biofilm on electrodes. In previous reports, we demonstrated that surface conditions significantly differ from those only hundreds of microns distal from the electrode surface. These included several 100 mV changes in redox potential, several units of pH changes, complete consumption of oxygen, and formation of hydrogen peroxide (Babauta et al., 2012b, 2013). Termed “microscale gradients” due to the spatial dimensions of such physicochemical gradients, microscale gradients related to biofilms on electrodes have only been studied recently. The relationship between these microscale gradients and electrode processes play an important role in understanding how energy transfer is possible at such long distances (Erable et al., 2012; Malvankar and Lovley, 2012; Snider et al., 2012; Renslow et al., 2013; Vargas et al., 2013). Here, we extend previous considerations of microscale gradients above electrodes to the use of polarized microelectrodes where similar current densities are expected to promote similar redox gradients.

We chose mats derived from Hot Lake as a model microbial mat system. Hot Lake is an epsomitic lake located near Oroville in north-central Washington that contains a benthic, phototrophic mat that exhibits seasonal variations in salinity, temperature, and light (Anderson, 1958; Lindemann et al., 2013). As the Hot Lake mat is exposed to the greatest sulfate concentrations of any described phototrophic mat and its potential for elevated electron flow mediated by reduced sulfur compounds was correspondingly high, this mat was an ideal system in which to study local electron transfer rates. Furthermore, previous reports have shown that Hot Lake mats harbor substantial populations of phylotypes consistent with sulfur cycling, indicative of high energy transfer. In this work, we quantify redox potential, pH and dissolved oxygen profiles in the field during day and night to demonstrate microscale gradients and demonstrate electricity generation using a mat fuel cell. We further quantify local electron transfer rates with an anodically polarized carbon microelectrode tip and analyze the microelectrode-associated community generated in comparison to the neighboring mat community. Finally, we propose a local electron transfer mechanism operating at the microelectrode tip to explain the differences between microelectrode-associated and neighboring mat communities.

## MATERIALS AND METHODS

### COLLECTING MICROBIAL MAT SAMPLES

Microbial mat samples for both field and laboratory experiments were collected from Hot Lake, Oroville, WA, USA (48.973062°N, 119.476876°W at an elevation of ~576 m) in July 2012 and May 2013, respectively. Mat was gently removed with 1–2″ of sediment at depth and was transferred into containers holding Hot Lake water.

### FIELD MICROBIAL MATS AND DEPTH PROFILES OF REDOX POTENTIAL, pH, AND OXYGEN

**FIGURE 1:**
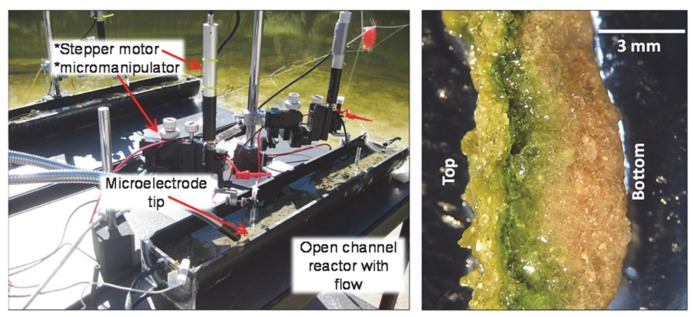
**Photo of open channel reactors containing microbial mat samples used in microprofiling (left).** Stereomicroscope image of excised mat slice on its side showing stratification (right).

For field experiments, microbial mat samples were placed in a 2-L open channel reactor and Hot Lake water pumped from the lake was circulated (~5 h^-^^1^) continuously during field measurements. Redox potential, pH, and dissolved oxygen microelectrode measurements were carried out as shown in **Figure 1.** Microelectrode movements were controlled by a Mercury Step motor controller PI M-230.10S Part No. M23010SX (Physik Instrumente, Auburn, MA, USA). Each microelectrode was positioned ~2000 μm above the mat surface and stepped down in 5 μm increments using custom microprofiler software. A Zeiss Stemi 2000 stereomicroscope was used to determine the locations of the microelectrode tip and surface of the mat. Redox potential, pH, and dissolved oxygen microelectrodes were constructed according to Lewandowski and Beyenal (2014).

### LABORATORY MICROBIAL MATS

For lab-scale experiments, microbial mat samples were transferred to our laboratory at Washington State University, Pullman, WA, USA. All samples were placed in reactors mimicking lake conditions without flow. First, sediment having approximately 5 cm thickness was added to the bottom of the reactor. Then, mat samples of 3–5 mm thickness and morphologically representative of neighboring mat were added to the top of the sediment. These mats were morphologically consistent with the mat described by Lindemann et al. (2013). During lab-scale experiments, deionized water was added periodically to maintain water level and salinity in the reactor. These mat samples were incubated in an enclosed growth chamber at ambient temperature that was artificially lighted using a Reeflux 250W 12000 K double ended metal halide lamp. The light was cycled in a 14 h on/10 h off cycle.

#### Electrochemical measurements inside laboratory microbial mats

**FIGURE 2:**
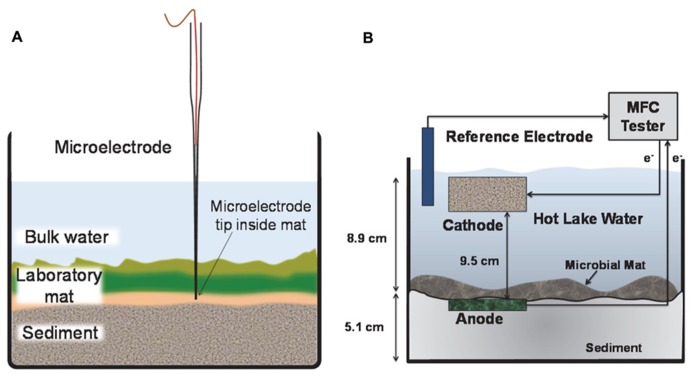
**Side view of laboratory mat incubation experiments.** **(A)** The microelectrode tip was positioned at depth inside the mat. The microelectrode was continuously polarized anodically against a Ag/AgCl reference electrode. **(B)** The anode of the mat fuel cell was placed directly below the mat and the cathode was placed in the bulk solution. The electrons are transferred from anode to cathode via an external circuit.

Electrochemical measurements refer to the generation of current from either anodic or cathodic reactions using anodic microelectrode polarization or a mat fuel cell. **Figure 2** shows the set up used for a microelectrode deployed inside the microbial mat (**Figure 2A**) and a mat fuel cell (**Figure 2B**). The microelectrode tip was positioned inside the laboratory mat at depths where physicochemical gradients (i.e., redox potential, pH, oxygen, S^2^^-^) were steep. Typically, this occurred at depths of 2–3 mm from the mat surface. At depths >3–4 mm, gradients were typically less steep indicating proximity to the sediment layers underneath the mat, which is similar in trend to what is observed at the bulk/mat surface interface. The steepest gradients were always observed to span the mat thickness and we targeted all microelectrode measurements at these depths. For the mat fuel cell, however, the anode could only be placed directly under the mat and was expected to respond to the gradients that extended beyond the mat thickness or that were external to the mat.

#### Mat fuel cell as current collector from the microbial mat

We used graphite felt for both the anode and the cathode (HP Materials Solutions, Inc., Woodland Hills, CA, USA) and each had projected surface areas of 2.14 × 10^-^^2^ and 6.48 × 10^-^^3^ m^2^, respectively. The electrodes were connected to a MFC Tester (Dewan et al., 2010) using a Grade 2, 0.635 mm diameter Ultra-Corrosion Resistant Titanium Wire (McMaster-Carr, Los Angeles, CA, USA) and 20 gauge copper insulated hookup wire. Ti wires were woven into the graphite felt and secured with nylon bolts. A mechanical and solder connection to the copper wire was sealed with silicone rubber to prevent water intrusion. The resistance of the copper wire, Ti wire and graphite felt connections were <1 Ohm at every point measured on each electrode.

The external circuit used to calculate power was a MFC Tester and was identical to the one described previously (Dewan et al., 2010). Briefly, the main components included a capacitor, two *n*-channel MOSFET switches to control energy storage and use, a USB-1608FS data acquisition module (Measurement Computing Corporation, Norton, MA, USA) and a PC with custom LabVIEW VI (National Instruments Corporation, Austin, TX, USA) to monitor and control the charge/discharge cycle. The generated energy was dissipated by shorting the capacitor through the ground of the data acquisition module thereby preventing electrons from being returned to the mat fuel cell. Power generation was monitored continuously by measuring the charge/discharge rates of the MFC Tester capacitor. We used Vc 50 mV and Vd 350 mV as charge and discharge potentials of the 1 F capacitor.

#### Microelectrodes as current collector from the microbial mat

Needle-type microelectrodes with 15–20 μm tip diameters were used to collect current inside the mat at depth. As described earlier for the field experiments, microelectrode tips were moved precisely inside laboratory mats using a micromanipulator with automated stepper motor and stereomicroscope. Once in position, a Gamry Interface 1000TM potentiostat (Gamry^®^ Instruments, Warminster, PA, USA) was used to control the microelectrode tip potential. A saturated Ag/AgCl reference electrode and platinum auxillary electrode completed the three-electrode setup (not shown in **Figure 2A**). Polarizing at +400 mV_Ag/AgCl_, electrons were passed from the microelectrode tip to the auxiliary electrode and the current was measured. Because the microelectrode tip was polarized above the open circuit potential to +400 mV_Ag/AgCl_, it acted as an anode inside the mat and was therefore unable to reduce oxygen. Thus the generally negative effects associated with oxygen reduction (i.e., generation of hydrogen peroxide and/or oxygen radicals) were avoided.

Procedures for the microelectrode used here are described in detail elsewhere (Lewandowski and Beyenal, 2014). Carbon fiber wires having 30 μm diameter (World Precision Instrument^©^) were used to construct microelectrodes. Corning 8161 glass was used to make a shaft for the carbon fiber. Carbon fibers were sealed in the glass by heat pulling. The carbon tip of the microelectrode was exposed by grinding away the glass seal using a diamond grinding wheel (Narishige, Model #EG-4). The diameter of the tip decreased after pulling to 15–20 μm due to the applied heat.

### COMMUNITY ANALYSIS

#### DNA extraction

Genomic DNA from accumulated biomass on the microelectrode tip and a 1.5 mm-thick region of neighboring mat biomass from near the tip were extracted for community analysis. Before extraction, samples were washed twice with filter-sterilized (0.2 μm) Hot Lake water collected from the aquarium followed by two washes with TE buffer (10 mM Tris pH 8.0, 1 mM EDTA pH 8.0). This assured that only firmly adherent cells remained at the tip of the microelectrode. DNA was extracted using QIAamp Kit (QIAGEN) by following manufacture’s instruction. Extracted DNA products were quantified prior to sequencing using a NanoDrop.

#### PacBio sequencing

Fragments of 16S rRNA genes containing variable V1–V3 regions were amplified from the extracted DNA with primers 27F (GAGTTTGATCMTGGCTCAG) and 515R (TTACCGCGGCTGCTGGCAC) (Kroes et al., 1999). Three barcodes, FB2 (TCATGAGTCGACACTA), FB8 (CTGCTAGAGTCTACAG), and RB2 (GCGATCTATGCACACG) were added to primers in paired asymmetric mode (FB2-RB2 for microelectrode sample and FB8-RB2 for microbial mat sample) for further sorting of each sample from pooled PacBio sequencing outcomes. Each PCR reaction was performed in duplicate 25 μl reactions containing 30 ng of DNA, 1X GoTaq@Flexi Buffer, 1.25 mM of MgCl_2_, 0.2 mM each dNTP, 0.1 μM of each barcoded primer (IDT), and 1.25 U of *Taq* polymerase (Promega). A C1000 Touch^TM^ Thermal Cycler (BIO-RAD, CA, USA) was used for the PCR as following program: (i) an initial denaturation step at 95°C for 4 min, (ii) 25 amplification cycles (95°C for 30 min, 57°C for 10 s, and 72°C for 20 s), and (iii) final extension at 72°C for 5 min. After this PCR amplification, the amplicons were purified (QIAGEN PCR purification kit) and quantified (NanoDrop). Amplicons were pooled and sequenced using a PacBio-RSII sequencer (Washington State University, Pullman, WA, USA). PacBio FASTAQ formatted circular consensus sequences (CCS) were processed and analyzed using Mothur v.1.32 (Schloss et al., 2009).

#### Sequence processing and analysis

Sequences were quality trimmed using a sliding window of 10 bp and an average quality score of 40 and sequences with one or more ambiguous bases were removed. Filtered sequences were dereplicated and aligned to SILVA-based bacterial reference alignment to which the aligned Hot Lake mat near full length sequences had been added as previously described (Lindemann et al., 2013). Sequences were then screened to remove those that did not align to positions 1044–10,241 of the reference alignment, filtered to remove non-informative columns, pre-clustered to >99% identity (allowing four differences), and dereplicated. Chimeras were identified and removed using UCHIME as implemented in mothur 1.32 in self-referential mode. Filtered sequences were then classified using a Wang approach against the RDP training set v.9 reference with 80% bootstrap cutoff and sequences of unknown classification were removed. Sequences were subsampled (to the size of the smallest group *n* = 10,423 sequences) and clustered into operational taxonomic units (OTUs) at 0.03 average distances using the average neighbor algorithm in mothur. OTUs were classified based upon the sequence classifications described above. Alpha and beta diversity metrics were calculated using subsampled sequences described above.

## RESULTS AND DISCUSSION

Traditionally, microbial mat communities have been studied using chemical sensors to determine fluxes of carbon, nitrogen, sulfur, and oxygen. From a practical engineering perspective, the flux of these elements creates an elemental balance that could be related to an equivalent flux of electrons if the governing redox reactions are known. Energy transformations can be studied directly and *in situ*. In order to establish the initial conditions for detection of electron transfer occurring at depth in the Hot Lake mat samples, we measured depth profiles of redox potential, pH, and oxygen around the diel cycle. These profiles were taken in explanted mat in open-flow channels continuously drawing lake water from the same depth in the lake as the collected mat. In addition, we measured depth profiles with similar trends in the laboratory (results not shown).

### REDOX POTENTIAL, pH, AND DISSOLVED OXYGEN DEPTH PROFILES

**FIGURE 3:**
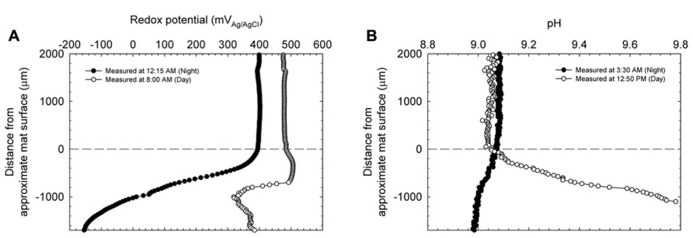
**Redox potential (A) and pH **(B)** depth profiles entering the upper strata of the explanted microbial mats at two time points during a diel cycle.** Zero indicates the approximate location of the mat’s surface.

**Figures 3A,B** show redox potential and pH depth profiles entering from the top of the mats at two time points during a 24-h day/night cycle and represent the cyclical response of the Hot Lake mat to light availability. During the day, redox potential remained positive at all depths in the mat from a bulk value of ~475 mV_Ag/AgCl_ to an observed minimum of ~320 mV_Ag/AgCl_. Redox potential reached a minimum at a depth of 1000 μm in the mat, and began to increase with further depth. During the night, the bulk value was slightly decreased to ~+400 mV_Ag/AgCl_ but sharply decreased to an observed minimum of approximately -150 mV_Ag/AgCl_. Unlike the profile observed during the day, the night redox gradient showed a continual decrease in redox potential, reaching a minimum at 1700 μm under the surface of the mat. **Figure 3B** shows a similar change in the pH trend in the top layers of the mat between gradients taken during the day and night. With a stable bulk pH of ~9.08 for both day and at night, we observed a maximum intra-mat pH of 9.8 during the day and a decrease to a pH of 8.98 during the night.

**FIGURE 4:**
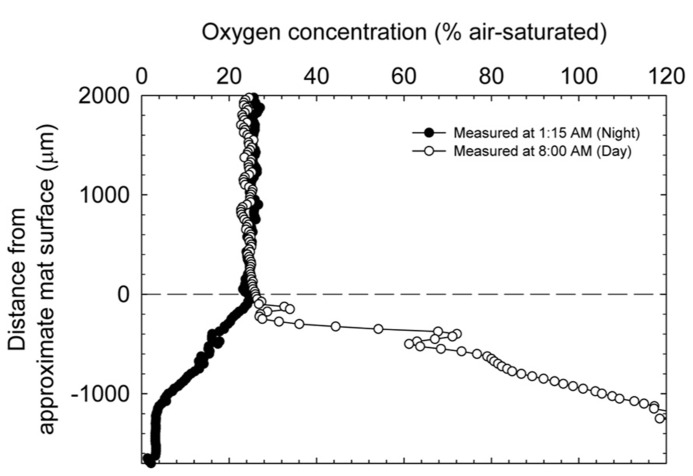
**Dissolved oxygen depth profiles entering the upper strata of the explanted microbial mats at two time points during a diel cycle**.

**Figure 4** shows the change in oxygen concentration in the top layers of the mat taken during the day and night and confirms the trends observed in redox potential and pH changes in the mat. Consistent with previous reports on cyanobacterial mat communities, oxygen concentration changed dramatically over the diel cycle. During the day, oxygen reached supersaturation at ~100 μM (headspace ~21% O_2_) and during the night was essentially undetectable.

During the day, oxygen saturated the top layers of the mat and likely controlled redox potential (cf. **Figures 3A** and **4**). However, the dip in redox potential in the mat, although still in the oxic range (Timothy et al., 2011), is not easily explained. Since steep physicochemical gradients span the mat, it is possible that unknown redox-active compounds slightly affect the primarily oxygen-dominated redox potential. At night, in the absence of light energy, reduced carbon is likely turned over until oxidant is depleted. Fermentation products then control redox potential at depth where oxidant (especially, oxygen) is depleted. Because fermentation occurs in highly reducing environments, redox potential at night is expected to decrease with depth in the mat. Consistent with this interpretation, oxygen concentration inside the mat at night decreased with depth, eventually to below the detection limit (**Figure 4**). The changes in pH likely follow changes in alkalinity with carbon dioxide equilibrium, including carbonate precipitation, during the diel cycle (Lindemann et al., 2013). pH closely mimics the dissolved oxygen curve as a spatial indication of photosynthesis in the upper strata of the mat. During the night, CO_2_ is regenerated in the mat by respiration with oxygen (at the top), sulfate, and also by fermentation. Following the gradient of fermentation and sulfate reduction, pH decreases from top to bottom in the mat.

### PHYSICOCHEMICAL GRADIENTS AND ELECTRON TRANSFER

Translating redox potential gradients into electron transfer rates could be achieved through the use of a mat fuel cell. The mat fuel cell harnesses electrons from the mat system externally using macro-scale electrodes and the power generation with the diel cycle is monitored. This is similar to sediment microbial fuel cells as have been extensively described (Reimers et al., 2006); however, instead of burying the anode in the sediment, we placed it directly below the mat. We should note that inserting electrodes into the mat ecosystem is difficult and induces modest disruption of existing mats. In the following sections, we demonstrate utilizable electron transfer inside the mat and that selectively drawing off electrons intra-mat may cause changes in the local mat community.

#### Mat fuel cell power generation

**FIGURE 5:**
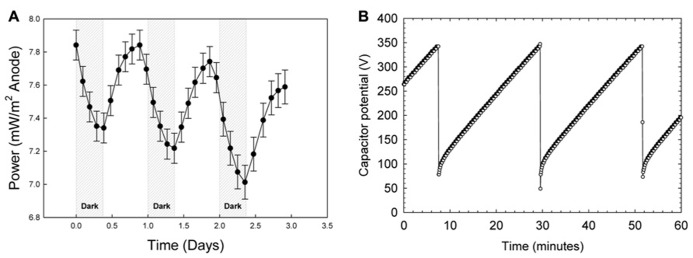
**(A)** Power generation by the mat fuel cell showing oscillations in tandem with the diel cycle where maximum power occurred near the end of each light cycle and reached a minimum at the end of the dark cycle. **(B)** An example charge/discharge cycle. The shorter charging time corresponds to higher power generation.

**Figure 5** shows power generation by a mat fuel cell. Power oscillated in tandem with the diel cycle where maximum power occurred near the end of each light cycle and reached a minimum at the end of the dark cycle. Each data point in **Figure 5A** corresponds to a charge/discharge cycle. The potential variation for an example charge discharge cycle is shown in **Figure 5B**. Because power was calculated by measuring the rate of charge/discharge of the MFC Tester, the cyclical nature of the measured power from the mat fuel cell directly translates to increases and decreases in the rate of charge/discharge. As the cathode was placed in the bulk and bulk oxygen concentration was nearly identical over the diel cycle (**Figure 4**), the majority of the oscillation was primarily localized to the anode directly below the mat. This suggests that the shift in gradients internal to the mat as discussed earlier and the gradients that extended outside the mat caused the power to oscillate.

Interestingly, observing increasing power in the presence of light is counter-intuitive given the trend in oxygen availability within the mat. As shown in **Figure 4**, oxygen concentration in the mat during the day reaches supersaturation and therefore should cause the anode directly under the mat to “discharge” as electrons are siphoned off to oxygen reduction, especially because the anode open circuit potential is only sustainable in the *absence* of oxygen. Admittedly, the contradiction is perplexing; however, we have shown previously that for mixed species environmental biofilms, oxygen gradients inside the biofilm can be quite sharp (Babauta et al., 2013). In that work, oxygen concentration depth profiles (using the same technique described here) showed that oxygen concentration could increase to nearly double the bulk concentration approximately 300 μm below the biofilm surface and subsequently decrease to undetectable levels within 2 mm. Considering that concentrations of oxygen and other metabolically relevant compounds reach maximal concentrations inside the mat and not necessarily at the base of the mat provides a valid reason as to why intra-mat processes cannot be fully explained by power cycling of the mat fuel cell. It is possible that the mat is not uniformly oxic and instead retains anoxic regions even in the presence of light; this is suggested by both the attenuation of wavelengths used for oxygenic photosynthesis and a dramatic increase in phylotypes known to employ microaerobic and anaerobic metabolisms below a depth of 2.5-3 mm in the Hot Lake mat (see **Figures 4** and **7**, Lindemann et al., 2013). Similar behavior was observed in several reports on phototrophic microbial fuel cells where current output in the first month primarily increased during light exposure but after 5 months decreased during light exposure (He et al., 2009). The authors attributed the change in trends to the dynamic interactions between photosynthetic microorganisms and heterotrophic bacteria. With the same reasoning, the complex interactions and energy transfer between dominant communities at different depths in the mat may cause the transient response of the mat fuel cell. Exactly which dominant community the anode is responding to is not determinable from **Figure 5** and only broad interpretations can be given. It is also important to note that Lindemann et al. (2013) documents seasonal changes in the mat community that were driven predominantly by changes in light availability. Such changes were hypothesized to rely upon the balance between photosynthetic production and heterotrophic consumption, especially in the diverse bottom layers of the mat.

#### New method to scale down to micro-scale electron transfer analysis

**FIGURE 6:**
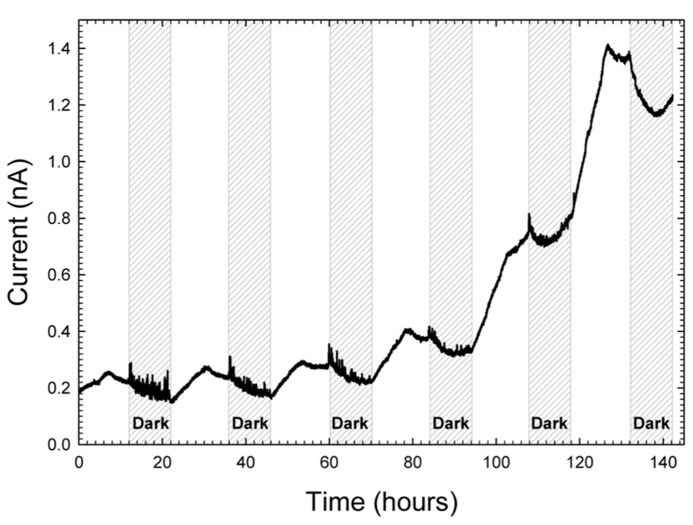
**Increase in current at a microelectrode tip placed 3 mm inside mat and polarized at +400 mV_Ag/AgCl_**.

To alleviate issues with scale and study local electron transfer, we scaled down from large electrodes to very precise microelectrodes. As shown in **Figure 2A**, microelectrodes were positioned such that the microelectrode tip (active surface) was placed 3 mm deep in the mat each time and were polarized to +400 mV_Ag/AgCl_. **Figure 6** shows that current generally increased during the day and decreased overnight. However, unlike in **Figure 5**, the maximum current was observed halfway through the light exposure period whereas for the mat fuel cell it was towards the end of the light exposure period. One possible explanation for the shift in current maximum was the proximity of the microelectrode tip to the oxygenic phototrophs at the top of the mat. Initially, inside the mat, reduced carbon compounds accumulated in the early stages of light exposure are oxidized vigorously by heterotrophs, which cause the increase in current (van der Meer et al., 2005; Behrens et al., 2008). Midway through the light cycle, oxygen concentration driven by oxygenic phototrophs, which should increase in magnitude and breadth during light cycles, began to influence the region surrounding the microelectrode tip and subsequently decrease the current. The results from this technique suggest that the observation of the maximal current within the light exposure period may depend greatly upon the depth at which the microelectrode tip is placed, which aligns well with the conclusions by He et al. (2009).

Surprisingly, these current oscillations were minor in comparison to the increase in baseline current. From a baseline of 0.2 nA, current increased over ~5 days to a maximum current of 1.4 nA. We note that with a microelectrode tip diameter of 20 μm, assuming hemispherical shape, the surface area was 6.28 × 10^-^^6^ cm^2^. Therefore, current densities were on the order of ~200 μA/cm^2^. The observed range of current density at the microelectrode tip was of the same order of magnitude as macro-scale investigations where we measured electrochemical gradients above polarized electrodes (Babauta et al., 2012b, 2013). Therefore, large and experimentally meaningful electrochemical gradients were imposed upon tightly localized regions in the mat. Broadly speaking, the baseline current increase reveals that more than one mechanism of energy transfer could be occurring at varying depths in the mat. Since the oscillations with the diel cycle are visible as a superposition on top of the increasing baseline, the increasing baseline itself is likely a result of a local change in the physicochemical environment in response to the electrochemical gradient formed around the microelectrode tip. Due to the complexity of the mat community, any number of explanations could explain the current increase. One possible explanation could be that the current increase is caused by a change in the local mat community.

### MICROBIAL COMMUNITY ANALYSIS

To determine whether the microelectrode-associated community was substantially different than the neighboring community, we extracted gDNA from the microelectrode tip and the surrounding mat after 27-day in-mat incubation, performed 16S rRNA sequencing and compared the two communities. We recovered about 0.33 μg of genomic DNA from the microelectrode tip. PacBio sequencing of the V1–V3 region of the 16S gene yielded a total of 66,811 CCS from both samples. Approximately 85% of the raw reads passed the PacBio quality filtering standards with an average length of 400 bp. Post-sequencing quality filtration and subsampling yielded 20,846 sequences (10,423 from each sample).

**FIGURE 7:**
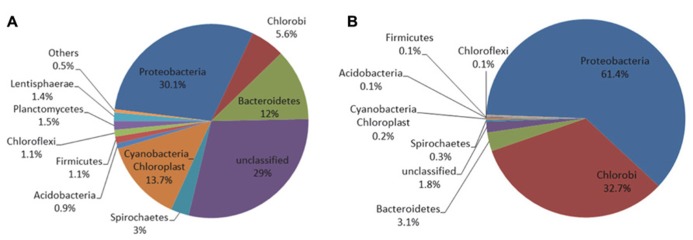
**Distribution of phyla for the microbial mat sample (A) and microelectrode tip sample (B)**.

**Figure 7A** shows that the microbial mat sample was dominated by members of the phyla *Proteobacteria* (30.1%), *Cyanobacteria*-*Chloroplast* (13.7%), *Bacteroidetes* (12%), *Chlorobi* (5.6%) and a large fraction of reads that could not be assigned to a phylum (29%). Except for *Chlorobi*, these phyla were also dominant in the Hot Lake mat throughout the seasonal cycle of 2011 (Lindemann et al., 2013). On the microelectrode tip, shown in **Figure 7B**, >95% of the reads were placed within only two phyla, *Proteobacteria* (61.4%) and *Chlorobi* (32.7%). In contrast with the neighboring mat, reads attributed to *Cyanobacteria-Chloroplast* and *Bacteroidetes* represented only about 0.2 and 3.1% of the total microelectrode-associated population, respectively. The microelectrode-associated community exhibited reduced alpha diversity (inverse Simpson metric = 8.462) compared with the neighboring mat (inverse Simpson metric = 48.351), and both species richness and evenness were lower in the microelectrode-associated community than the neighboring mat. The distance between the two samples in Bray-Curtis beta diversity, which compares the relative abundances of species observed between communities, was 0.892. The divergence exhibited between the two communities suggests that a specific subpopulation of mat organisms was enriched on the microelectrode tip.

Sequences were clustered into OTUs and the dominant bacterial OTUs from the microelectrode-associated community are listed in **Table 1**. With one exception (OTU ME1), the other dominant bacterial OTUs in the table displayed low abundance in the neighboring mat community. OTU ME1, whose nearest cultured neighbor is *Prosthecochloris aestuarii* DSM 271 (97% identity across the sequenced region, GenBank accession: NR_074364.1), accounted for 4.6% of reads in the neighboring mat community, but more than 30% of the total reads from the microelectrode-associated community*.* OTU ME2 is very closely related (99%) to *Loktanella vestfoldensis* NBRC 102487 (GenBank accession: AB681826.1), known to be a strictly aerobic bacterium. Similarly, OTU ME4 and OTU ME6 were also identified as aerobic species which belong to genera *Catellibacterium* and *Thiomicrospira*, respectively. The remaining dominant OTUs in *Rhodobacteraceae* could not be classified below the family level.

**Table 1:** Most abundant OTUs in the microelectrode-associated community.

Dominant OTUs	Family	Genus	Relative abundance (%)	
			Neighboring mat	Microelectrode
ME1	*Chlorobiaceae*	*Prosthecochloris*	4.59	30.34
ME2	*Rhodobacteracea*e	*Loktanella*	0.14	9.40
ME3	*Rhodobacteraceae*	Unclassified	0.09	9.17
ME4	*Rhodobacteraceae*	*Catellibacterium*	0.51	7.35
ME5	*Rhodobacteraceae*	Unclassified	0.12	4.88
ME6	*Piscirickettsiaceae*	*Thiomicrospira*	0.18	1.97

The members of genus *Prosthecochloris* are known to be obligately anaerobic phototrophic green sulfur bacteria and can utilize sulfide and elemental sulfur as electron donors for photosynthesis (Gorlenko, 2001; Imhoff, 2003). The final oxidation product is soluble sulfate (Imhoff, 2003). The high relative abundance of OTU ME1 in the neighboring mat community and its prevalence on the microelectrode-associated community may be due to the abundance of sulfide in the Hot Lake microbial mat. Several previous studies have also reported that anaerobic phototrophic bacteria can generate electrical current in MFCs (Xing et al., 2008; Nishio et al., 2010). However, it remains unclear whether this dominant ME1 *Prothecochloris* bacterium is involved in current generation from our polarized microelectrode (**Figure 6**). In addition, OTU ME6 is classified within *Thiomicrospira*, which is also known to be chemolithoautotrophic with reduced sulfur compounds such as thiosulfate, elemental sulfur and sulfide as electron donors to reduce oxygen molecules (Brinkhoff et al., 1999; Takai et al., 2004). It is possible that these microorganisms were enriched because the microelectrode tip happened to be placed precisely at a depth in the mat that frequently contains both trace amounts of oxygen and sulfide or cycles between oxic and sulfidic conditions, which is underscored by phylogenetic evidence of a transition in metabolism from aerobic to microaerobic or anaerobic at equivalent depth in the mat (Lindemann et al., 2013). However, the microelectrode-associated community contains organisms with seemingly incompatible metabolisms. *Loktanella* members, for example, known to be strict aerobes (Van Trappen et al., 2004), were significant members of the community alongside *Prosthecochloris*. The curious co-enrichment of the strictly anaerobic *Prosthecochloris* and strictly aerobic *Loktanella, Catellibacterium,* and *Thiomicrospira* on the microelectrode tip requires further investigation integrating electrochemical function with activity of isolated members.

**FIGURE 8:**
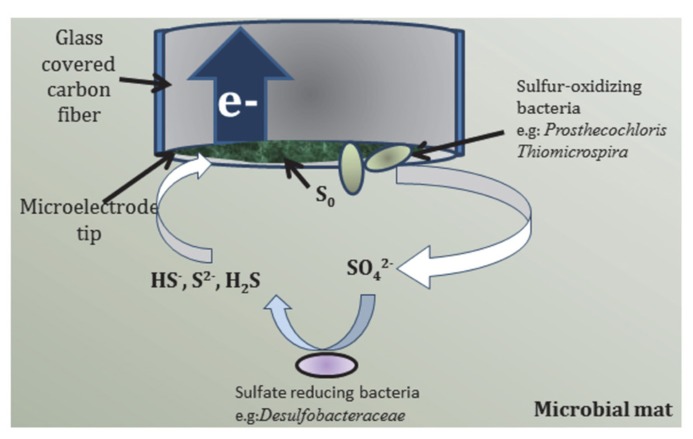
**Proposed pathway of bioelectrochemical cycling of sulfur species at the microelectrode tip**.

Recent studies have called into question the utility of PacBio sequencing for 16S rRNA analysis of microbial communities due to its error rate (Mosher et al., 2013); therefore, our quality filtration of raw sequences was extremely strict. As Mosher et al. (2013) did not report any quality filtration post-sequencing for PacBio reads, it is not possible to directly compare the stringency of our approach or our final error rate post-filtration to theirs. If the increased error rate of PacBio did proliferate clusters that are, in reality, derived from the same parent sequences in the communities, abnormally high alpha diversity is expected as each real OTU is, essentially, counted multiple times (Mosher et al., 2013). However, our inverse Simpson metric for the mat community was 48.351, which is similar to the Hot Lake mat at an equivalent depth as assayed using Itags (cf. **Figure 8**; Lindemann et al., 2013) and much lower for the microelectrode-associated community (8.462). Therefore, we did not detect abnormally high species richness in our communities, suggesting that our quality filtration was stringent enough to account for the error rates associated with PacBio in our comparative analysis.

### COMBINING SULFIDE ELECTROCHEMISTRY WITH MICROBIAL COMMUNITY ANALYSIS

Because of its importance to environmental remediation of sulfide-contaminated industrial waste, sulfide electrochemical detection has been well-studied on carbon-based electrodes (Lawrence et al., 2004). According to Lawrence et al. (2004), HS^-^ is oxidized electrochemically to elemental sulfur on the electrode surface and acts as a passivation layer. Therefore, on unmodified carbon electrodes, sulfide oxidation to elemental sulfur is an unstable process with decreasing current as passivation proceeds and follows the half reaction listed in **Table 2**. Although the standard reduction potential is negative, anodic current is typically observed at more positive potentials on unmodified carbon electrodes due to a high overpotential and irreversibility of sulfur redox systems (Bard et al., 1985). Lawrence et al. (2004) observed sulfide oxidation to occur at approximately +200 mV_Ag/AgCl_ in 50 mM phosphate buffer at pH 7.4 on bare glassy carbon surface. At pH 10, that value would shift to approximately +274 mV_Ag/AgCl_. To a first approximation then, the polarization of the carbon fiber microelectrode tip at +400 mV_Ag/AgCl_ would likely oxidize any HS^-^ diffusing to the microelectrode tip to elemental sulfur. Considering this, the microelectrode tip should have exhibited decreasing current due to sulfur passivation because Hot Lake mats are known to generate sulfide in the dark which is also verified with microelectrode measurements (data not shown). However, the increase in current during light periods cannot be explained electrochemically.

**Table 2:** Selected reduction reactions of relevant sulfur compounds (taken from Bard et al., 1985).

Type	Standard half reaction	Standard reduction potential V vs. Ag/AgCl(sat)
Sulfide to sulfur	HS^-^ → S+2e^-^ +H^+^	-0.259
Polysulfide to sulfide*	$S_{2}^{2-}$ +2e^-^ +2H^+^ → 2HS^-^	0.090
	$S_{3}^{2-}$ +4e^-^ +3H^+^ → 3HS^-^	-0.100
Sulfate to sulfite	${SO}_{4}^{2-}$ +2e^-^ +4H^+^ → H_2_ SO_3_ +H_2_ O	-0.039
Tetrathionate to thiosulfate	$S_{4}O_{6}^{2-}$ +2e^-^ → ${2S}_{2}O_{3}^{2-}$	-0.117
Thiosulfate to sulfide*	$S_{2}O_{3}^{2-}$ +8e^-^ +8H^+^ → 2HS^-^ +3H_2_ O	0.021

*Occurs through multiple electron transfer steps (Schippers, 2004).

Elemental sulfur cannot be electrochemically oxidized by the microelectrode tip and, therefore, accumulates on the surface. The relative abundance of *Prosthecochloris* at the microelectrode tip suggests that the accumulated elemental sulfur may be oxidized to soluble thiosulfate $\left(S_{2}O_{3}^{2-}\right)$ and sulfate $\left({SO}_{4}^{2-}\right)$ biotically if the region surrounding the microelectrode is persistently anoxic (Imhoff, 2003; Muyzer and Stam, 2008; Sun et al., 2010; Liang et al., 2013). If biotic oxidation of elemental sulfur did occur at the microelectrode tip, then a reasonable hypothesis for increasing current during light exposure can be formulated. The hypothesized sulfur cycle is shown in **Figure 8** where green phototrophic sulfur bacteria (*Prosthecochloris*) and aerobic sulfur-oxidizer (*Thiomicrospira*) oxidize elemental sulfur to sulfate in the presence of light. Sulfate is then reduced back to HS^-^ biotically by sulfate-reducing bacteria (e.g., *Desulfobacteraceae*) present in the mat. The sulfur cycle, completed by the electrochemical oxidation of HS^-^ at the microelectrode tip, can occur indefinitely until HS^-^ is depleted. Assuming that turnover of elemental sulfur by green phototrophic sulfur bacteria is the limiting step, enrichment of these members over time would increase the baseline current seen at the microelectrode tip. Furthermore, the inhibition of elemental sulfur turnover in the dark would also explain the oscillation of current. Therefore, the trend seen in **Figure 6** can be reasonably explained by the proposed bioelectrochemical cycling of sulfur.

The above discussion on sulfur cycling remains speculative because sulfur speciation in sediments is quite complex as the type of (metal) sulfide and solubility affects the mechanism of abiotic and biotic oxidation to sulfate (Schippers, 2004). Here, both the thiosulfate or polysulfide mechanisms of sulfide oxidation could account for the various sulfur species. Thiosulfate, sulfite, polythionates, polysulfides, and elemental sulfur are all possible intermediates that could play an important role in sulfur cycling in Hot Lake mats. Of equal importance is the availability of iron, oxygen, manganese, and nitrate (Jørgensen, 2004) in the mat at depth. Finally, the role of calcium sulfates and magnesium sulfates in Hot Lake (Lindemann et al., 2013) on sulfur cycling is unknown. The possibility of targeting a particular sulfur compound redox potential on the microelectrode is interesting in terms of the ability to select for sub-populations of heterotrophs existing only along steep physicochemical gradients inside the mat. Carefully selecting the polarization potential of the microelectrode would tune into specific redox reactions (i.e., sulfur) that are utilized by targeted heterotrophs. Inspection of several standard reduction potentials of sulfur reactions listed in **Table 2** suggests that we could electrochemically differentiate sulfur reactions and thereby select those microorganisms that could metabolize the resulting products. We also note that sulfur oxidation is not the only exploitable redox reaction, as polarizing the microelectrode ca. -200 mV_Ag/AgCl_ would tune into oxygen reduction and remove oxygen at the microelectrode surface. However, these electrochemical activities remain unexplored in Hot Lake microbial mats.

## CONCLUSION

We quantified physicochemical gradients (redox potential, pH, and dissolved oxygen) and their diel variations within the Hot Lake mat. We further employed a mat fuel cell to demonstrate that phototrophic microbial mats can generate electricity, which increases upon exposure to light. We also found that a microelectrode with a carbon tip can be used to study local electron transfer processes in a microbial mat. Anodically polarizing a microelectrode tip showed increased current with time, reaching a maximum during the day period of a day–night cycle. The increased current at the microelectrode tip indicated presence of electroactive compounds near the microelectrode tip. 16S rRNA analysis revealed that the bacterial community attached to the polarized microelectrode tip was distinct from that of the neighboring microbial mat. The reduced alpha diversity of the microelectrode-associated community and the large distance in beta diversity between this community and the neighboring mat, driven by differences in the relative abundances of reads attributed to phyla *Proteobacteria* and *Chlorobi*, suggested that the polarized microelectrode tip may locally enhance the growth of certain bacterial phylotypes. Furthermore, the characteristics of the most abundant OTUs in the microelectrode-associated community suggested that the current cycle obtained from our polarized microelectrode may be related to bacterial sulfur cycling. It remains unclear whether electron transfer to the electrode surface during current generation was direct or indirect. Our results describe a new method for monitoring of local electron transfer rates within a microbial mat and subsequent assaying of the adherent community developed at the microscale. This method can contribute to future electron transfer studies and aid in our ability to enrich specific subpopulations *in situ* within microbial mats.

## Conflict of Interest Statement

The authors declare that the research was conducted in the absence of any commercial or financial relationships that could be construed as a potential conflict of interest.
